# Now You See Them, Now You Don’t: Age Differences in Risk Aversion

**DOI:** 10.1093/geroni/igaa057.1176

**Published:** 2020-12-16

**Authors:** Barış Sevi, Jenna Wilson, JoNell Strough, Natalie Shook

**Affiliations:** 1 University of Connecticut, Storrs, Connecticut, United States; 2 West Virginia University, Morgantown, West Virginia, United States

## Abstract

Older age has often, but not always, been associated with greater risk aversion. Some have suggested that age differences in risk may reflect age-related declines in cognitive abilities. This study investigated the robustness of age differences in risk aversion across three different risk-taking measures, after controlling for cognitive abilities. Community-dwelling younger (n = 75; 25-36 years, M age = 29.01) and older (n = 74; 60-90 years, M age = 69.11) adults completed self-report and behavioral measures of risk aversion and several measures of cognitive abilities. Results showed that older adults reported significantly greater risk aversion than young adults on the behavioral measure of risk (Balloon Analogue Risk Task, BART), but not on the self-report measures (Framing Task and Choice Dilemmas Questionnaire). Greater risk aversion on BART was significantly associated with lower analytic thinking, slower processing speed, and worse shifting of attention. Therefore, we tested the relation between age and risk aversion on the BART while controlling for these three cognitive abilities. Age differences in risk aversion remained significant even after accounting for cognitive abilities. Our results suggest that the lack of consistent age differences in risk aversion in the literature may at least partly be due to measurement differences, which raises concerns about the construct validity of these measures of risk aversion. Moreover, cognitive decline may not explain age differences in risk. Further research is needed to understand factors that dampen and heighten risk aversion in people of diverse ages.

